# Supplementary material for: A force-sensitive mutation reveals a non-canonical role for dynein in anaphase progression
Source: J Cell Biol. 2024 Jul 1;223(10):e202310022. doi: 10.1083/jcb.202310022 (PMC11215527; doi:10.1083/jcb.202310022)
Supplement: Table S1 — shows occurrence in simulations of hydrogen bond pairs involving S3386. [file JCB_202310022_TableS1.docx]

**Table S1. Occurrence in simulations of hydrogen bond pairs involving S3386.**

| **Donor (position)** | **Acceptor (position)** | **Occurrence (%) ^(1)^** |
| --- | --- | --- |
| Ser3386 (loop) | Val3382 (helix 6) | 61.33 |
| Cys3389 (CC2) | Ser3386 (loop) | 41.24 |

(1) 3.6 µs of all-atom MD simulations
