# Supplementary material for: A force-sensitive mutation reveals a non-canonical role for dynein in anaphase progression
Source: J Cell Biol. 2024 Jul 1;223(10):e202310022. doi: 10.1083/jcb.202310022 (PMC11215527; doi:10.1083/jcb.202310022)
Supplement: Table S2 — shows occurrence in simulations of hydrogen bond pairs involving C3386. [file JCB_202310022_TableS2.docx]

**Table S2. Occurrence in simulations of hydrogen bond pairs involving C3386**.

| **Donor (position)** | **Acceptor (position)** | **Occurrence (%) ^(1)^** |
| --- | --- | --- |
| Cys3386 (loop) | Val3382 (helix 6) | 69.20 |
| Cys3389 (CC2) | Cys3386 (loop) | 32.32 |

1. 3.6 µs of all-atom MD simulations
