# Supplementary material for: A force-sensitive mutation reveals a non-canonical role for dynein in anaphase progression
Source: J Cell Biol. 2024 Jul 1;223(10):e202310022. doi: 10.1083/jcb.202310022 (PMC11215527; doi:10.1083/jcb.202310022)
Supplement: Table S3 — shows occurrence in simulations of hydrophobic interaction partners of C3386. [file JCB_202310022_TableS3.docx]

**Table S3. Occurrence in simulations of hydrophobic interaction partners of C3386.**

| **Partner (position)** | **Occurrence (%) ^(1)^** |
| --- | --- |
| Val3307 (helix 1) | 99.94 |
| Met3310 (loop between helices 1 and 2) | 100 |
| Pro3314 (loop between helices 1 and 2) | 99.80 |
| Val3317 (helix 2) | 100 |
| Val3382 (helix 6) | 96.76 |
| Ala3385 (loop between helix 6 and CC2) | 100 |
| Ala3388 (CC2) | 100 |
| Cys3389 (CC2) | 100 |

1. 3.6 µs of all-atom MD simulations
