# Supplementary material for: A force-sensitive mutation reveals a non-canonical role for dynein in anaphase progression
Source: J Cell Biol. 2024 Jul 1;223(10):e202310022. doi: 10.1083/jcb.202310022 (PMC11215527; doi:10.1083/jcb.202310022)
Supplement: Table S4 — shows oligonucleotides used to create Dhc mutations in Drosophila. [file JCB_202310022_TableS4.docx]

**Table S4. Oligonucleotides used to create Dhc mutations in *Drosophila*.**

| ***Drosophila* mutation** | **Human equivalent** | **gRNA cloning oligos ^(1)^** | **Donor oligo ^(2)^** |
| --- | --- | --- | --- |
| *K129I* ^(3)^ | *K129I* | f: gtcgCGCGGCCTGGTCGTGGAGG  r: aaacCCTCCACGACCAGGCCGCG | TCACTTCACCAACAGTCGGATGGCATCTTTGGCCTGCATCAAGCGCGGCCTGGTCGTGGA**A**GC**C**GACAtcTCGATCCATTCGCAGCTGCGGCTGATCAACTTCTCGGACGGATCTCCCTACGAGACGCTG (s) |
| *F579Y* ^(4)^ | *F582Y* | f: gtcgGAAAATGCGGAACATCTCGT  r: aaacACGAGATGTTCCGCATTTTC | ATCAGCTGCGTCTGGTACTCCCGGATAGCGCCACGGATGTGCGGACGCACGAAGAGCGCATTGAAACGCGAGAAAATGCGG**T**ACATCTCGTTGGCGTTCTTGGCTGTTCCCAGCTGATCACGTAGATGAGCAGTTATGCGAGTCTCCACGCGAT (as) |
| *R1557Q* | *R1567Q* | f: gtcgGAATTCGGAGCTGATGCTT  r: aaacAAGCATCAGCTCCGAATTC | ACCTTGGGCGATTTGGTCACCTTCTTCATCAACCCAAGGAATTCGGAGCTGATGCTTTG**A**AA**TT**GCGAAGTCTCCACCGGAAGGAGCGTCTTAATATCAGCGCTGCCCGAGAAGATTCCCTCCAA (as) |
| *R1951C* ^(5)^ | *R1962C* | f: gtcgCGACGAGTTCAATCGACTGG  r: aaacCCAGTCGATTGAACTCGTCG | GGCCGAATCTTTGTCGGTCTGTGCCAGGTGGGCGCATGGGGCTGCTTCGACGAGTTCAAT**T**G**TT**TGGAGGAGCGTATGCTCTCCGCTTGCTCACAGCAAATCCAGACCATTCAGGAGGCGCTGA (s) |
| *K3226T* | *K3241T* | f: gtcgCATCTGCTTAAGCTTGGCGT  r: aaacACGCCAAGCTTAAGCAGATG | CTGAATTTCCTGCGATTGAATCTTCTTCTTCTCGGCCTCCTGCTGATCCTGGAACATCTG**TG**T**T**A**AT**TTGGCGTTGGCAGCCTCGTTCTTGGCCTGCAACTCCTGCTTCTTCACAGCCAGCGACTTT (as) |
| *R3370Q* ^(6)^ | *R3384Q* | f: gtcgAAGGTTAATCGCGCCAGTA  r: aaacTACTGGCGCGATTAACCTT | GTATCAAAAAACAGCGATGATGTTCGAGAGAAGATGAAGTCCAAATATCTGAGCAATCCGGACTATAACTTCGAGAAGGTTAATC**AA**GC**G**AGTATGGCGTGTGGTCCTATGGTAAAATGGGCCATTG (s)^(7)^  CGATGATGTTCGAGAGAAGATGAAGTCCAAATATCTGAGCAATCCGGACTATAACTTCGAGAAGGTTAATC**AA**GC**G**AGTATGGCGTGTGGTCCTATGGTAAAATGGGCCATTGCTCAGGTAAGATTTATATCGAATTGATCGTGAATAG (s) |
| *S3372C+*  *C3375S* | *S3386C+*  *C3389S* | f: gtcgAAGGTTAATCGCGCCAGTA  r: aaacTACTGGCGCGATTAACCTT | GTATCAAAAAACAGCGATGATGTTCGAGAGAAGATGAAGTCCAAATATCTGAGCAATCCGGACTATAACTTCGAGAAGGTTAATCGCGCC**T**GTATGGCG**A**GTGGTCCTATGGTAAAATGGGCCATTG (s) |
| *C3375S* | *C3389S* | f: gtcgAAGGTTAATCGCGCCAGTA  r: aaacTACTGGCGCGATTAACCTT | GTATCAAAAAACAGCGATGATGTTCGAGAGAAGATGAAGTCCAAATATCTGAGCAATCCGGACTATAACTTCGAGAAGGTTAATCGCGC**T**AG**C**ATGGCG**A**GTGGTCCTATGGTAAAATGGGCCATTG (s) |
| *H3808P* ^(8)^ | *H3822P* | f:gtcgTCTTTAGCGAGTACTGGTAG  r:aaacCTACCAGTACTCGCTAAAGA | GGTTATTATACAGCACCGTGGAGAAGATATCGAGGAACATCTTTAGCGAGTACTGGTAGAG**A**AAG**G**GCACCTGGTTCAGACTGTCCATGGTGAAGTAGATGTTGCTGCACGCCACAGAAAGTGGGA (as) |

1. f, forward; r, reverse. Sequences corresponding to target sequence are upper case; sequences used for cloning of the annealed oligo pairs into the gRNA plasmid pCFD3 are lower case
2. Donors correspond to either the sense (s) or antisense (as) strand, as indicated. Positions that differ from the equivalent region of the target genome are shown in bold. In cases where a disease mutation does not interfere with recutting by the Cas9/gRNA complex, synonymous mutations in the seed sequence or protospacer adjacent motif were also introduced
3. The *Dhc^null^* allele was also recovered in this experiment
4. Corresponds to the mouse *Loa* mutation, which is F580Y in the orthologous Dync1h1 protein
5. The *L1952K* allele was also recovered in this experiment
6. The *S3372C* allele was also recovered in this experiment. The same gRNA plasmid was used to make the *S3372C + C3375S* and *C3375S* alleles
7. 2 different donors, which are shifted with respect to each other in the 5′ to 3′ direction, were injected into separate batches of embryos. Each donor led to recovery of both *R3370Q* and *S3372C* alleles
8. The *ΔL3810+Y3811* and *Y3811F* alleles were also recovered in this experiment
