# Supplementary material for: A force-sensitive mutation reveals a non-canonical role for dynein in anaphase progression
Source: J Cell Biol. 2024 Jul 1;223(10):e202310022. doi: 10.1083/jcb.202310022 (PMC11215527; doi:10.1083/jcb.202310022)
Supplement: Table S5 — shows primary antibodies used in this study. [file JCB_202310022_TableS5.docx]

**Table S5. Primary antibodies used in this study.**

| **Target** | **Host** | **Catalog number / Source ^(1)^** | **RRID ^(2)^** | **Dilution for IF / IB ^(3)^** |
| --- | --- | --- | --- | --- |
|  |  |  |  |  |
| α-Tubulin | Mouse | T6199 (clone DM1A) / Sigma Aldrich | AB_477583 | 1:500 / NA |
| α-Tubulin | Rabbit | ab208752 / Abcam | AB_869989 | NA / 1:2000 |
| α4-Tubulin | Rabbit | NA / Fahmy et al., 2014 | AB_2567589 | 1:400 / NA |
| BubR1 | Rabbit | NA / Logarinho et al., 2004 | - | 1:1000 / NA |
| Centrosomin | Sheep | NA / Lucas and Raff, 2007 | - | 1:500 / NA |
| Cyclin B | Rabbit | NA / Whitfield et al., 1990 | - | 1:500 / NA |
| Cysteine string protein | Mouse | ab49 / Developmental Studies Hybridoma Bank (DSHB) | AB_2307340 | 1:250 / NA |
| Dynein heavy chain | Mouse | 2C11-2 / DSHB | AB_2091523 | NA / 1:1000 |
| Mad2 | Rabbit | NA / Caous et al., 2015 | - | NA / 1:1000 |
| p150^Glued^  (C-term) | Rabbit | NA / Kim et al., 2007 | - | NA / 1:10,000 |
| Phospho-histone H3 | Rabbit | 9701 / Cell Signaling | AB_331535 | 1:200 / NA |
| Spindly | Rabbit | NA / Griffis et al., 2007 | - | 1:1000 / NA |
| Synaptotagmin | Rabbit | NA / West et al., 2015 | - | 1:1000 / NA |
|  |  |  |  |  |

1. NA, not applicable
2. If available
3. IF, immunofluorescence; IB, immunoblotting; NA, not applicable
