# Supplementary material for: A force-sensitive mutation reveals a non-canonical role for dynein in anaphase progression
Source: J Cell Biol. 2024 Jul 1;223(10):e202310022. doi: 10.1083/jcb.202310022 (PMC11215527; doi:10.1083/jcb.202310022)
Supplement: Table S6 — shows secondary antibodies used in this study. [file JCB_202310022_TableS6.docx]

**Table S6. Secondary antibodies used in this study.**

| **Target** | **Host** | **Catalog number / Source ^(1)^** | **RRID ^(2)^** | **Dilution for IF / IB ^(3)^** |
| --- | --- | --- | --- | --- |
| Donkey anti-mouse IgG (H+L), Alexa488 |  | A-21202 / ThermoFisher Scientific | AB_141607 | 1:500 / NA |
| Donkey anti-Rabbit IgG (H+L), Alexa555 |  | A-31572 / ThermoFisher Scientific | AB_162543 | 1:500 / NA |
| Donkey anti-Sheep IgG (H+L), Alexa647 |  | A-21448 / ThermoFisher Scientific | AB_2535865 | 1:500 / NA |
| Ms m-IgGκ BP-HRP |  | sc-516102 / Santa Cruz | AB_2687626 | NA / 1:10,000 |
| anti-Rabbit IgG, HRP-linked whole antibody |  | NA934 / Cytiva | AB_772206 | NA / 1:10,000 |

1. NA, not applicable
2. If available
3. IF, immunofluorescence; IB, immunoblotting; NA, not applicable
