# Supplementary figures and images for: A force-sensitive mutation reveals a non-canonical role for dynein in anaphase progression
Source: J Cell Biol. 2024 Jul 1;223(10):e202310022. doi: 10.1083/jcb.202310022 (PMC11215527; doi:10.1083/jcb.202310022)

anti-Dhc

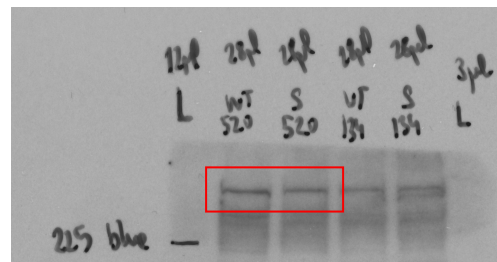

anti- $\alpha$ 1-Tubulin

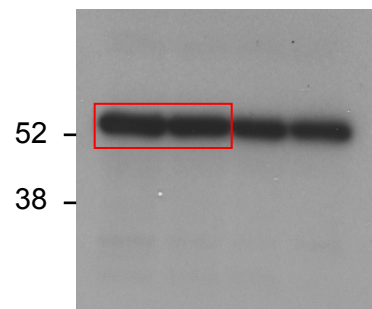

Supplement: SourceData F2 — is the source file for Fig. 2. [file JCB_202310022_SourceDataF2.pdf]

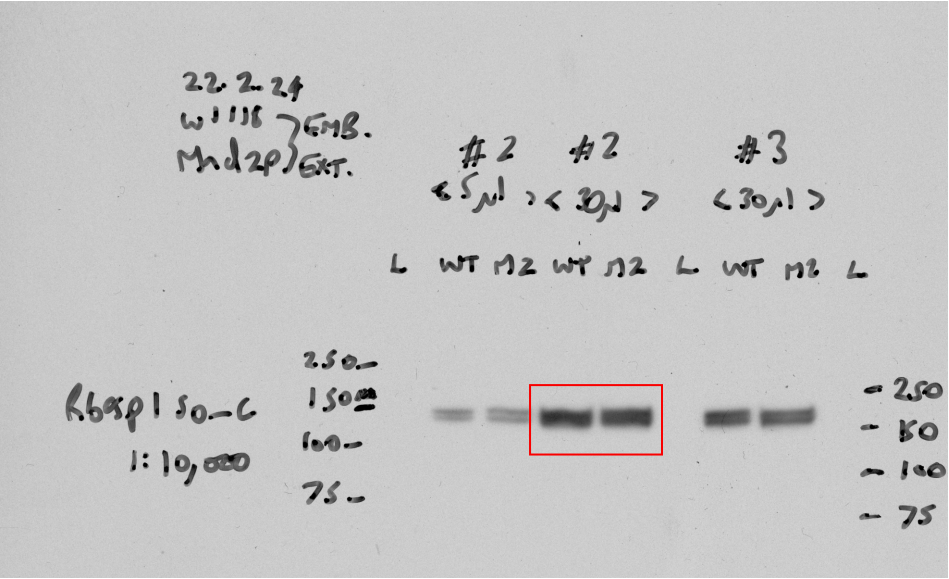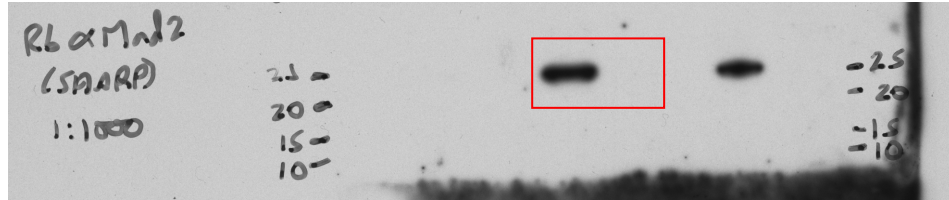

Supplement: SourceData F8 — is the source file for Fig. 8. [file JCB_202310022_SourceDataF8.pdf]

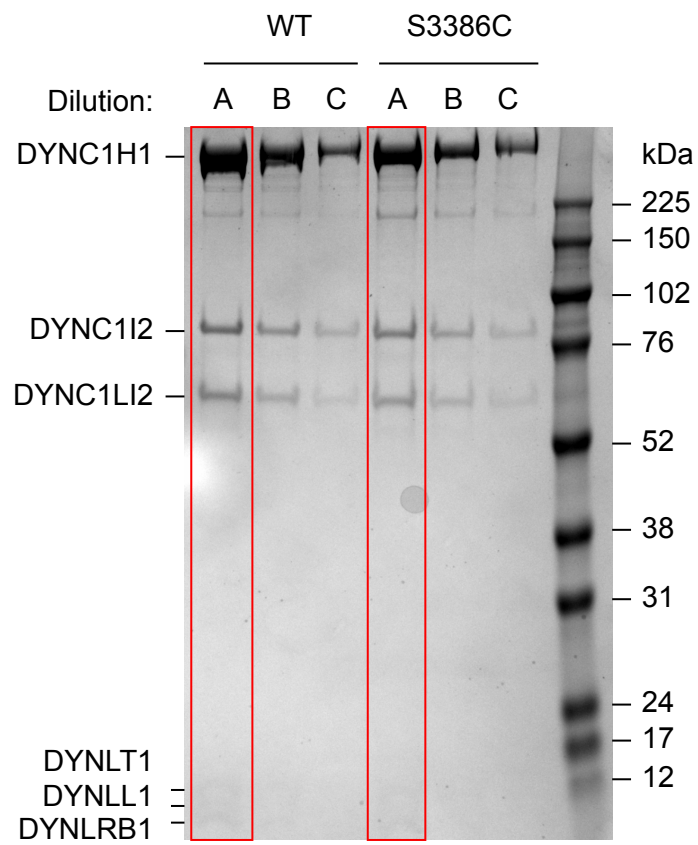

Supplement: SourceData FS5 — is the source file for Fig. S5. [file JCB_202310022_SourceDataFS5.pdf]
